# Characterization of the First Marine Pestivirus, Phocoena Pestivirus (PhoPeV)

**DOI:** 10.3390/v17010107

**Published:** 2025-01-14

**Authors:** Lars Söder, Denise Meyer, Olaf Isken, Norbert Tautz, Matthias König, Alexander Postel, Paul Becher

**Affiliations:** 1 Institute of Virology, Department of Infectious Diseases, University of Veterinary Medicine Hannover, D-30559 Hannover, Germany; lars.soeder@tiho-hannover.de (L.S.); alexander.postel@tiho-hannover.de (A.P.); 2 Institute of Virology and Cell Biology, University of Lübeck, D-23562 Lübeck, Germany; olaf.isken@uni-luebeck.de (O.I.); norbert.tautz@uni-luebeck.de (N.T.); 3 Institute of Virology, Faculty of Veterinary Medicine, Justus-Liebig-University, D-35392 Giessen, Germany; matthias.koenig@vetmed.uni-giessen.de

**Keywords:** Phocoena pestivirus, Bungowannah pestivirus, classical swine fever virus, *Flaviviridae*, innate immunity, pestivirus, cell tropism, CD46, DNAJC14, harbor porpoise

## Abstract

The first marine pestivirus, Phocoena pestivirus (PhoPeV), isolated from harbor porpoise, has been recently described. To further characterize this unique pestivirus, its host cell tropism and growth kinetics were determined in different cell lines. In addition, the interaction of PhoPeV with innate immunity in porcine epithelial cells and the role of selected cellular factors involved in the viral entry and RNA replication of PhoPeV were investigated in comparison to closely and distantly related pestiviruses. While Bungowannah pestivirus (BuPV), a unique porcine pestivirus closely related to PhoPeV, exhibits a broad cell tropism, PhoPeV only infects cells from pigs, cattle, sheep, and cats, as has been described for classical swine fever virus (CSFV). Viral titers correlate with the amount of intracellular PhoPeV-specific RNA detected in the tested cell lines. PhoPeV replicates most efficiently in the porcine kidney cell line SK6. Pestiviruses generally counteract the cellular innate immune response by degradation of interferon regulatory factor 3 (IRF3) mediated by the viral N-terminal protease (N^pro^). No degradation of IRF3 and an increased expression of the type 1 interferon-stimulated antiviral protein Mx1 was observed in porcine cells infected with PhoPeV whose genome lacks the N^pro^ encoding region. Infection of a CD46-deficient porcine cell line suggested that CD46, which is implicated in the viral entry of several pestiviruses, is not a major factor for the viral entry of PhoPeV. Moreover, the results of this study confirmed that the cellular factor DNAJC14 plays a crucial role in viral RNA replication of non-cytopathic pestiviruses, including PhoPeV.

## 1. Introduction

The virus family *Flaviviridae* inherits a large number of important pathogens for human and animal health. This virus family is subdivided into the four genera *Orthoflavivirus*, *Hepacivirus*, *Pegivirus*, and *Pestivirus*, with over 90 different species [1]. The genus *Pestivirus*, comprises a variety of pathogens infecting farm animals and wildlife [2]. Until the year 2000, there were only four approved species within the genus *Pestivirus*: bovine viral diarrhea virus-1 (BVDV-1, new species name: *Pestivirus bovis*), bovine viral diarrhea virus-2 (BVDV-2, new species name: *Pestivirus tauri*), classical swine fever virus (CSFV, new species name: *Pestivirus suis*), and border disease virus (BDV, new species name: *Pestivirus ovis*) [3]. To date, 19 pestivirus species are known, demonstrating their broad host range, which extends across species boundaries of the order *Artiodactyla*, including a growing number of new host species in the orders *Chiroptera*, *Rodentia*, and *Pholidota* [4].

A few years ago, the first marine pestivirus, Phocoena pestivirus (PhoPeV, species *Pestivirus M*) was described, which expands the host range of pestiviruses to the marine ecosystem [4,5,6]. PhoPeV was first discovered in harbor porpoises (*Phocoena phocoena*) of the Dutch North Sea [5] and later in animals of the Baltic Sea [6]. Interestingly, harbor porpoises belong to the order *Cetacea*, which is proposed to be paraphyletic to the order *Artiodactyla*, which includes a large number of pestivirus host species [7]. So far, the pathogenesis of PhoPeV is unknown, as no pathological lesions were directly associated with PhoPeV infection in harbor porpoises [5,6]. PhoPeV was detected in different organs of harbor porpoises (e.g., lung, brain, liver, kidney, spleen, and reproductive organs), which suggests a systemic infection [5,6]. Infections with other pestiviruses like CSFV and BVDV can result in acute diseases, immunosuppression, or inapparent infections [2,8]. Moreover, intrauterine infections of the fetus can lead to abortions, fetal malformations, and stillbirth or to development of immunotolerance against the virus, resulting in persistently infected (PI) offspring, which were mostly described in cases of infection related to BVDV [2,9,10]. These PI animals are infected for their entire lives and shed the virus continuously [2]. Phylogenetic analyses showed that PhoPeV is most closely related to Bungowannah pestivirus (BuPV, species *Pestivirus australiaense*) and Linda virus (LindaV, species *Pestivirus* L.) [4,5]. BuPV and LindaV have caused local epidemic outbreaks on farms in Australia and Austria, with severe and fatal consequences after the infection of pregnant sows [11,12,13]. After intrauterine infection, affected piglets experienced sudden death associated with porcine myocarditis syndrome during BuPV infection [14] or congenital tremor (CT) with lateral shaking when infected with LindaV [13].

Pestiviruses are small enveloped positive-sense single-stranded (ss)RNA viruses. The pestivirus genome is about 12.3 kb in size and exhibits one large open reading frame (ORF) encoding a single polyprotein of approximately 3900 amino acids (aa). In comparison to other members of the *Flaviviridae*, pestiviruses express two unique viral proteins involved in overcoming antiviral defense by interacting with the host innate immune response, the N-terminal autoprotease (N^pro^), and the structural glycoprotein E^rns^ [15,16]. Interestingly, PhoPeV lacks the N^pro^ coding region, resulting in the expression of a shorter pestiviral polyprotein comprising 3762 aa. N^pro^ has been shown to directly counteract the host innate immunity by mediating proteasomal degradation of interferon regulatory transcription factor 3 (IRF3) and interference with IRF7 in plasmacytoid dendritic cells (pDCs) [15,17,18], thereby directly preventing type 1 alpha/beta interferon (IFN) production [18]. The glycoprotein E^rns^ functions as a viral structural protein but is also partially secreted from infected cells [19]. E^rns^ is characterized by ribonuclease (RNase) activity and binds viral ss and double-stranded (ds)RNA to cleave it. The resulting reduction of viral genomic RNA, which represents a pathogen-associated molecular pattern (PAMP), inhibits the cellular innate immune response [16,19,20,21]. Both viral proteins have an important impact, as viral innate immune antagonists, on the establishment of persistent infections [22].

By their effects in tissue culture cells, two different biotypes, the non-cytopathic (ncp) and cytopathic (cp) biotype of pestiviruses such as BVDV, can be distinguished [10,23,24,25]. Only ncp pestiviruses are able to establish persistent infections after intrauterine infection of the fetus [26,27]. PI animals shed large amounts of the virus and play a major role in the transmission and spread of pestiviruses [10]. The cp pestiviruses emerge in PI animals by RNA recombination and can cause fatal mucosal disease in these animals [23,27,28]. Persistent infection by ncp BVDV strains is maintained primarily through control of viral RNA replication mediated by regulation of the non-structural (NS) protein 2 autoprotease activity and NS2-3 cleavage by the host factor DNAJC14. In contrast, efficient NS3 expression of cp pestivirus strains is independent of cellular DNAJC14 [27,29]. The dependence for efficient viral RNA replication on DNAJC14 has also been confirmed for other pestivirus species, including CSFV and BuPV [29]. In contrast, ncp atypical porcine pestivirus (APPV, species *Pestivirus scrofae*) replicates DNAJC14 independently [29,30]. In cell culture, PhoPeV exhibits an ncp phenotype [5,6].

In order to extend the knowledge about PhoPeV, which so far is the only pestivirus known to lack N^pro^, host cell tropism, growth kinetics in different cell lines, and interaction of PhoPeV with innate immunity in porcine epithelial cells were studied. Furthermore, the role of the cellular factors CD46 and DNAJC14 in viral entry and RNA genome replication was analyzed.

## 2. Materials and Methods

### 2.1. Cells and Viruses

For the analysis of viral cell tropism and in vitro replication, different mammalian cell lines were used (Table 1). Since BuPV is genetically closely related to PhoPeV, we referred to published data on BuPV when selecting the cell lines [31]. To investigate whether the replication of PhoPeV is DNAJC14-dependent, SK6 DNAJC14 knockout (SK6 DNAJC14-KO), as well as complemented knockout cells (SK6 DNAJC14-KO GST-Jiv90-WT), were infected with PhoPeV [29].

The cell lines were grown in Dulbecco’s Modified Eagle’s medium (EDulb; Gibco Thermo Fisher, Waltham, MA, USA) or in minimum essential medium (MEM; Gibco Thermo Fisher, Waltham, MA, USA) containing antibiotics, supplemented with 5% or 10% horse serum (HS) or fetal bovine serum (FBS) (Table 1). FBS batches were tested regularly for the absence of pestivirus genome by reverse transcription (RT)-PCR and for the absence of neutralizing antibodies against BVDV. The SK6 DNAJC14-KO, and complemented SK6 DNAJC14-KO GST-Jiv90-WT cell lines were cultured in MEM supplemented with 5 mg/mL of Puromycin and 1 mg/mL of G418 (GST-Jiv90-WT).

In the present study, the previously described PhoPeV isolate from the Baltic Sea was used [6]. The CSFV strain Alfort-Tübingen (Alfort-T) was obtained from the CSF virus collection of the EU and WOAH Reference Laboratory for CSF (Institute of Virology, University of Veterinary Medicine, Hannover, Germany), and the BuPV isolate was kindly provided by Peter Kirkland (Elizabeth Macarthur, Agriculture Institute, Menangle, NSW, Australia). The APPV isolate L277 has been described previously [32].

### 2.2. Antibodies and Antisera

PhoPeV antigen was detected by the monoclonal antibody (MAb) WB166 [33], kindly provided by Helen Crooke (Animal and Plant Health Agency, New Haw, Surrey, UK). For the detection of BuPV, a porcine polyclonal BuPV-specific antiserum was used [12], kindly provided by Peter Kirkland (Elizabeth Macarthur, Agriculture Institute, Menangle, NSW, Australia). The MAb BVD/C16 was applied for the detection of CSFV Alfort-T [34]. Antigen of APPV was detected by using a porcine APPV-specific antiserum [35]. Secondary antibodies were Cy™3-affiniPure goat anti-mouse IgG (H+L) and Cy™3-affiniPure goat anti-pig IgG (H+L).

For detection of IRF3 and Mx1 expression, a rabbit anti-porcine IRF3 serum (kindly provided by Nicolas Ruggli, Institute of Virology and Immunology IVI, Mittelhäusern, Switzerland) and anti-Mx1 MAb M143 (kindly provided by Georg Kochs, Institute of Virology, University of Freiburg, Freiburg, Germany) were used and detected by a polyclonal rabbit anti-mouse immunoglobulin/HRP (Dako A/S, Denmark) or a goat anti-rabbit IgG H&L (HRP) (Abcam, Cambridge, UK), respectively [36,37]. For the detection of beta-actin, a monoclonal beta-actin antibody was used (Thermo Fisher, Waltham, MA, USA).

### 2.3. Cell Tropism Analysis of PhoPeV

In vitro cell tropism was examined by infection of twelve different mammalian cell lines with PhoPeV, BuPV, and CSFV Alfort-T. Briefly, the cell lines SK6, PK-15, 38A_1_D, MDBK, SFTR, SEK-2B, CRFK, Vero, BHK-21, Balb3T3, HEK-293T, and HeLa (Table 1) were infected using a multiplicity of infection (MOI) of 0.5 for 1 h at 37 °C and 5% CO_2_. Cells incubated with growth medium only served as a negative control. Consecutively, inoculum was removed, and the cells were washed three times with phosphate-buffered saline (PBS) and incubated with growth medium for 72 h at 37 °C and 5% CO_2_. Supernatants were sub-cultured on the same cell lines. At 72 hpi, cells were fixed by heat treatment for 3 h at 80 °C (first and second infection cycle) and evaluated by immunofluorescence (IF) staining.

### 2.4. Virus Growth in Cell Lines Susceptible to PhoPeV

To investigate the growth efficiency in different cell lines, SK6, PK-15, 38A_1_D, MDBK, SFTR, and CRFK cells were infected with PhoPeV. Cells were seeded in six-well plates, incubated with growth medium for 24 h, and then infected with PhoPeV using an MOI of 0.1. Subsequently, the cells were washed five times with PBS and incubated with growth medium for 96 h at 37 °C and 5% CO_2_. Cell culture supernatants were obtained immediately after washing and at 12, 24, 48, 72, and 96 hpi. The growth kinetics were determined in three independent experimental approaches. In addition, total cellular RNA was prepared at each sampling time point. For the SK6 cell line, three additional sampling time points (120, 144, and 168 hpi) were applied. At 96 or 168 hpi, the cells were fixed by heat treatment to control viral infection by IF staining.

#### 2.4.1. Virus Titration

The cell culture supernatants were titrated in quadruplicates by virus endpoint dilution assay on SK6 cells. After 72 h, cells were fixed by heat treatment and the viral titer determined by IF staining using the Spearman–Kärber method [38,39]. Statistical analyses were performed and illustrated using GraphPad Prism (GraphPad Software version 9.0.0, GraphPad Software, Inc., Boston, MA, USA).

#### 2.4.2. RNA Preparation and Real-Time Reverse Transcriptase PCR

RNA extraction from infected cells was performed using the NucleoSpin^®^ RNA kit according to the manufacturer’s description (MACHEREY-NAGEL GmbH & Co. KG, Düren, Germany). The intracellular RNA concentration was determined by spectrophotometric analysis using the NanoDrop™ 2000/2000c spectrophotometer (Thermo Fisher, Waltham, MA, USA). Each sample was adjusted to 10 ng RNA/µL in resuspension buffer. Viral RNA was analyzed in duplicates by quantitative RT-PCR (RT-qPCR) according to the previously published protocol and using the primers marinePV_204fw and marinePV_340rev that target a 137-base-pair (bp) fragment within the 5′-UTR of PhoPeV [6]. RT-qPCR was performed using the QuantiTect^®^ SYBR^®^ Green RT-PCR kit (Qiagen N.V., Venlo, The Netherlands) and the CFX96™ real-time system (Bio-Rad Laboratories, Inc, Hercules, CA, USA). Based on a PhoPeV-specific RNA standard, viral RNA copy numbers were calculated and illustrated using GraphPad Prism.

### 2.5. PhoPeV Replication in the Presence or Absence of DNAJC14 After Cellular Infection

To investigate the influence of DNAJC14 on in vitro replication of PhoPeV, SK6 wild-type (WT), SK6 DNAJC14-KO-, and SK6 DNAJC14-KO GST-Jiv90-WT cells were infected with PhoPeV in two independent experiments, respectively. In addition, these cells were infected with BuPV, CSFV Alfort-T, and APPV. Cells were infected at an MOI of 1 or 0.1 (APPV) and fixed by heat treatment at 72 hpi. Viral antigen was detected by IF staining.

### 2.6. Immunofluorescence Staining

Heat-fixed cells were rehydrated by adding PBS-0.05% Tween20. For antigen detection, the cells were incubated for 1 h at 37 °C or overnight at 4 °C (APPV) with the corresponding MAbs (PhoPeV = WB166, 1:500 dilution; CSFV = BVD/C16, 1:50 dilution) or antisera (BuPV antiserum = 1:12,000 dilution; APPV antiserum = 1:1000 dilution). Afterwards, the respective conjugates [Cy™3-affiniPure goat anti-mouse IgG (H+L) diluted 1:800 or Cy™3-affiniPure goat anti-pig IgG (H+L) diluted 1:250] were added and incubated for 1 h at 37 °C. Cell nuclei were stained with a 4′,6-Diamidino-2-phenylindol (DAPI; diluted 1:500) solution for 15 min at room temperature (RT) in the dark. All dilutions of antibodies or antisera were prepared in PBS-0.05% Tween20. After each incubation step, the cells were washed three times with PBS-0.05% Tween20.

Evaluation of the results was performed by IF microscopy using the Nikon Eclipse Ti (Nikon Corporation, Minato, Japan). Pictures and overlays were created using the NIS-Elements AR software (NIS-Elements AR version 5.21.03, Nikon Corporation, Minato, Japan).

### 2.7. Immunoblot Analysis

Porcine PK-15 cells were infected with PhoPeV, BuPV, and CSFV Alfort-T using a MOI of 0.5, respectively. At 24 and 48 hpi, the cells were washed three times with cold PBS and subsequently lysed by using NP40 lysis buffer [50 mM Tris/HCl (pH 7.5), 150 mM NaCl, 0.5 g sodium desoxycholate, 1 mL Nonidet P40 (NP40), 2 complete protease inhibitor pellets; AppliChem GmbH, Darmstadt, Germany], respectively. Total cellular protein levels were quantified using the Pierce^®^ BCA Protein Assay Kit (Thermo Fisher, Waltham, MA, USA) and the Tecan Sunrise Microplate Reader (Tecan, Männedorf, Switzerland). Proteins (50 μg per slot) were separated using 10% sodium dodecyl sulphate (SDS) gels under reducing conditions. Gel electrophoresis was performed in an SE250 Mighty Small II Mini Vertical Protein Electrophoresis Unit (Hoefer Inc., Bridgewater, MA, USA). The proteins were transferred to PVDF membranes by semi-dry immunoblot. The membranes were blocked overnight in a 5% powdered milk TBS-0.02% Tween20 blocking solution (ROTH, Karlsruhe, Germany).

For the detection of IRF3 and Mx1, the membranes were incubated with the respective primary antibody (Mx1 = M143, 1:1000 dilution; IRF3 = rabbit anti-porcine IRF3 serum, 1:4000 dilution), followed by incubation with the conjugates, polyclonal rabbit anti-mouse immunoglobulin/HRP (1:2000 dilution), or goat anti-rabbit IgG H&L (HRP) (1:40,000 dilution), respectively. All primary antibodies and serum dilutions, as well as the conjugate dilutions, were prepared in blocking solution. After each incubation step, the membrane was washed three times with TBS-0.02% Tween20. As loading control, the housekeeping protein beta-actin was detected after stripping of the membrane using the respective buffer [100 mM NaOH, 2% SDS and 0.5% DTT] at 55 °C for 1 h. For the detection of beta-actin, a monoclonal beta-actin antibody (1:1000 dilution) in combination with the described polyclonal rabbit anti-mouse immunoglobulin/HRP (1:2000 dilution) was used. Protein detection was performed using the ECL Select™ Western Blotting Detection Reagent Kit (Amersham™, Amersham, UK). Immunoblots were evaluated using the BioRad ChemiDoc™ MP Imaging System and Image Lab software (Image Lab version 6.1.0, BioRad Laboratories, Inc., Hercules, CA, USA).

## 3. Results

### 3.1. Host Cell Tropism of PhoPeV

**Figure 1:**
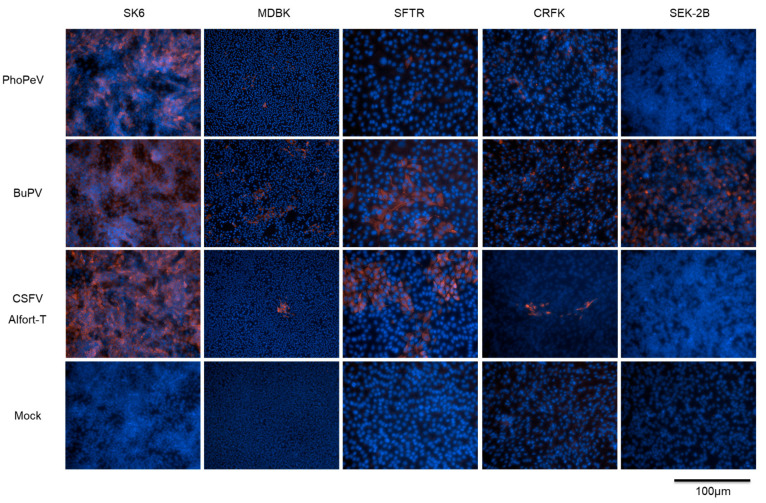
Infection of different mammalian cell lines with PhoPeV, BuPV, and CSFV Alfort-T. Swine kidney-6 (SK6), Madin–Darby bovine kidney (MDBK), sheep fetal thymus (SFTR), Crandel–Rees feline kidney (CRFK), and seal kidney cells (SEK-2B) were infected with PhoPeV, BuPV, and CSFV Alfort-T at an MOI of 0.5. Viral antigens were detected by immunofluorescence analysis at 72 hpi. Cell nuclei were stained with DAPI (blue). All mock controls (bottom) tested negative.

So far, the host range of PhoPeV has not been characterized in detail. Therefore, in vitro cell tropism studies of PhoPeV were performed in comparison to the pestiviruses BuPV and CSFV. PhoPeV is genetically more closely related to BuPV, which showed a broad in vitro cell tropism in contrast to the more different CSFV which grows mainly in porcine cell lines [31]. Twelve mammalian cell lines of different species (Table 1) were infected with PhoPeV, BuPV, and CSFV Alfort-T using an MOI of 0.5. Six out of twelve cell lines were permissive for PhoPeV, including porcine (SK6, PK-15, 38A_1_D), bovine (MDBK), ovine (SFTR), and feline (CRFK) cells, while phocine (SEK-2B), hamster (BHK-21), monkey (Vero), mouse (Balb3T3), and human (HeLa, HEK-293-T) cells were not susceptible to PhoPeV (Table 2). Approximately 70% of the cell layer of SK6 cells were infected with PhoPeV, while the infection rate on other porcine, bovine, ovine, and feline cells was lower (Figure 1). In contrast to other susceptible cell lines, PhoPeV was not detected in ovine cells after using the cell culture supernatant of the first infection cycle in SFTR cells (Table 2). Since seals represent marine mammals that might be in contact with harbor porpoises, the permanent harbor seal cell line SEK-2B was included in the present study. No PhoPeV infection was detected for these cells. However, BuPV replicates on these cells, as well as on BHK-21, Vero, Hela, and HEK-293T cells (Table 2).Theses results confirmed the previously reported broad cell tropism of BuPV [31].

Taken together, the results indicate a restricted host cell tropism of PhoPeV resembling the tropism of CSFV and show that the genetically close relatedness of PhoPeV to BuPV does not correlate with the broad species-specific cell tropism of BuPV.

### 3.2. Viral Growth and Viral RNA Production of PhoPeV in Different Cell Lines

**Figure 2:**
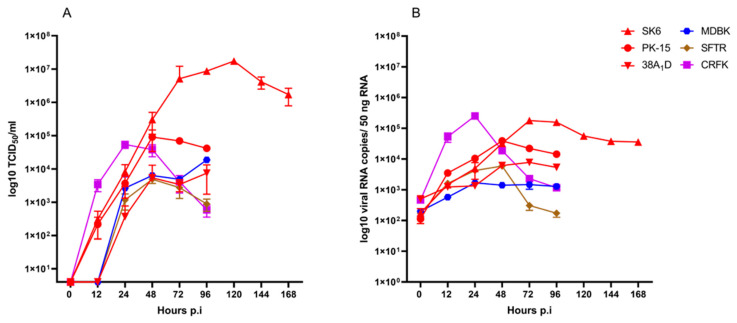
Viral growth kinetics and viral RNA synthesis in porcine, bovine, ovine, and feline cell lines infected with PhoPeV. Porcine SK6, PK-15, and 38A_1_D cells, bovine MDBK cells, ovine SFTR cells, and feline CRFK cells were infected with PhoPeV at an MOI of 0.1. Supernatants and cell lysates of infected cells were collected over a period of 96 hpi or 168 hpi (SK6). (**A**) Virus titers were determined as 50% tissue culture infectious doses (TCID_50_) per mL. (**B**) After extraction of total cellular RNA from the collected cell lysates, the viral RNA copy numbers were determined by RT-qPCR and expressed as log 10 copies per 50 ng of RNA. Each time point was evaluated in triplicates. Mean values and standard deviations were calculated by GraphPad Prism software version 9.0.0.

To further characterize the viral growth of PhoPeV on the permissive cell lines, SK6, PK-15, 38A_1_D, MDBK, SFTR, and CRFK cells were infected with PhoPeV using an MOI of 0.1. Cell culture supernatants and cell lysates were collected over a period of 96 hpi or, in the case of SK6 cells, for 168 hpi, respectively. Viral growth of PhoPeV was most efficient in the porcine cell line SK6, reaching a titer of 1.74 × 10^7^ TCID_50_/mL at 120 hpi (Figure 2A). In comparison, after infection of PK-15 cells, a maximum titer of 9.03 × 10^4^ TCID_50_/mL was detected at 48 hpi, while virus infection of the other cell lines resulted in lower titers (10^2^–10^4^ TCID_50_/mL).

The efficiency and kinetic of viral RNA synthesis was analyzed by RT-qPCR of total cellular RNA. After infection of the different cell lines with PhoPeV, viral RNA copy numbers were consistent with the infectious viral titers detected in the cell culture supernatant (Figure 2A,B). However, the highest viral RNA copy numbers were detected in CRFK cells at 24 hpi and in SK6 cells at 72 hpi. In contrast to the high viral titers detected in the supernatant of infected SK6 cells, the amount of viral RNA in these cells is comparable to the one detected in CRFK cells (Figure 2B). In accordance with the low virus titers detected in the supernatants of infected MDBK and SFTR cells, the RNA copy numbers ranged between 10^2^ and 10^4^ RNA copies/50 ng RNA (Figure 2B).

CD46 has been described as playing a key role in the cell entry of BVDV [40], while it is non-essential for CSFV and BuPV [35]. Due to the similar cell tropism of PhoPeV and CSFV and the genetically close relationship of PhoPeV to BuPV, it was interesting to analyze whether the absence of CD46 in the CD46-deficient porcine cell line 38A_1_D affects viral entry and replication of PhoPeV. After infection of 38A_1_D cells with PhoPeV, an infectious viral titer of 7.48 × 10^3^ TCID_50_/mL and RNA copy numbers of 5.32 × 10^3^/50 ng RNA were detected at 96 hpi (Figure 2A,B). This suggests that porcine CD46 does not play a major role in the viral entry and replication of PhoPeV.

### 3.3. Expression of IRF3 and the Mx Protein in Porcine Cells After Infection with PhoPeV

As a major difference from other pestiviruses, the viral genome of PhoPeV does not encode for the viral N-terminal protease N^pro^. This protein induces proteasomal degradation of IRF3, which leads to the prevention of IFN alpha/beta induction and inhibition of the expression of effector proteins encoded by interferon-stimulated genes (ISGs) such as Mx1 [41,42]. Accordingly, N^pro^ is significantly implicated in the inhibition of cellular antiviral defense against pestiviruses. With regard to the lack of the N^pro^ coding region in its genome, it is reasonable to speculate that PhoPeV cannot inhibit the type 1 IFN induction and expression of ISGs. To test this hypothesis, expression of IRF3 and the Mx1 protein were analyzed in porcine cells infected with PhoPeV by immunoblot. As it was previously reported that SK6 cells cannot be stimulated to produce type 1 IFN, PK-15 cells were used for these experiments [15,42]. As controls, CSFV Alfort-T and BuPV were included. Infection of PK-15 cells with the individual viruses was confirmed by IF and immunoblot analyses.

**Figure 3:**
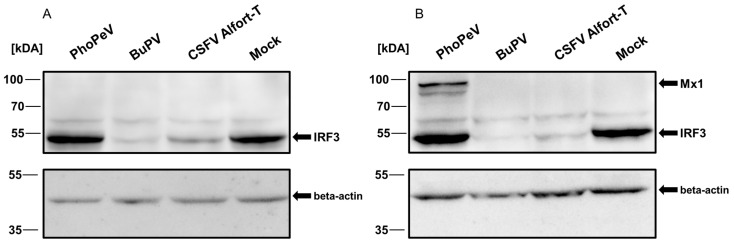
Expression of IRF3 and the Mx1 protein in porcine cells at 24 h (**A**, left) and 48 h (**B**, right) after infection with PhoPeV, BuPV, and CSFV Alfort-T. Porcine PK-15 cells were infected with PhoPeV, BuPV, and CSFV Alfort-T at an MOI of 0.5. Cell lysates were collected at 24 hpi and 48 hpi, and protein levels were quantified. For each lysate, 50 µg of total protein were used for immunoblot analysis, as witnessed by comparable beta-actin content in each lane (bottom panels). As a negative control, PK-15 cells were inoculated with cell culture medium (Mock). Degradation of IRF3 was detected after infection with BuPV and CSFV Alfort-T (**A**,**B**). PhoPeV-infected cells show no degradation of IRF3 (**A**,**B**) and induction of Mx1 expression (**B**).

In the lysates of non-infected PK-15 cells and cells infected with PhoPeV, a constant expression of a similar amount of IRF3 was detected at 24 and 48 hpi (Figure 3A,B). In contrast, after infection of PK-15 cells with BuPV and CSFV Alfort-T, reduced amounts of IRF3 were detected at 24 and 48 hpi (Figure 3A,B). Expression of Mx1 protein was detected only in cells infected with PhoPeV at 48 hpi but not in cells infected with BuPV and CSFV (Figure 3). Taken together, these results show that PhoPeV does not induce proteasomal degradation of IRF3, and consequently, this unique pestivirus (which lacks the N^pro^ encoding region) cannot inhibit a cellular type 1 IFN-mediated immune response in PK-15 cells.

### 3.4. RNA Replication of PhoPeV Is Dependent on DNAJC14

According to their growth characteristics in tissue culture cells, pestiviruses are divided into two biotypes: non-cytopathogenic (ncp) and cytopathogenic (cp) viruses. The majority of pestiviruses are ncp viruses. For different ncp pestiviruses such as BVDV, CSFV, and BuPV, it was shown that DNAJC14 is crucial for their viral RNA replication in vitro [29]. However, more recently, it was reported that APPV replicates independently from DNAJC14 [30].

**Figure 4:**
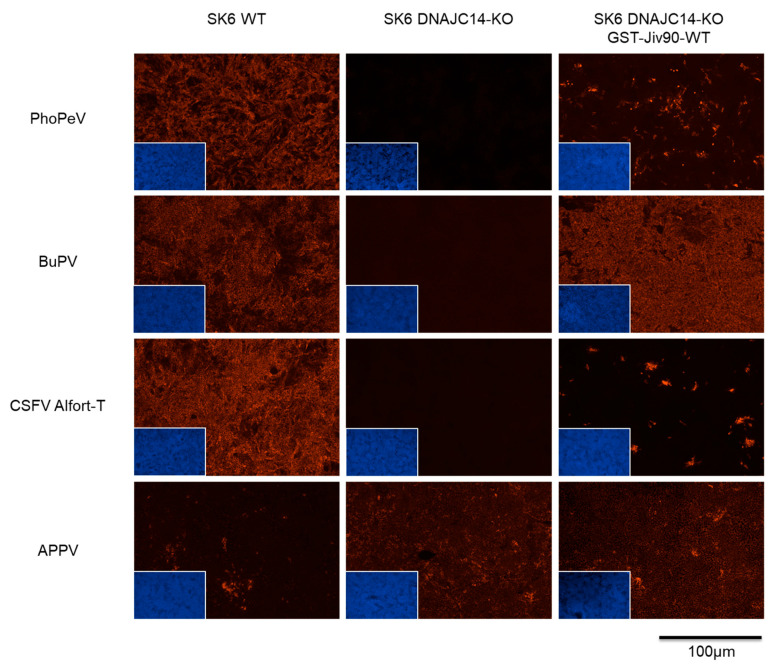
Relevance of the cellular protein DNAJC14 for viral RNA replication of PhoPeV. Cells were infected with PhoPeV, BuPV, and CSFV Alfort-T using an MOI of 1 and with APPV at an MOI of 0.1. Viral antigen was detected by immunofluorescence at 72 hpi. Virus infection of all selected viruses, in the presence of DNAJC14, was detected in the swine kidney 6 wild-type cell line (SK6 WT) and in the DNAJC14-KO rescue cells (SK6 DNAJC14-KO GST-Jiv90-WT). Similar to BuPV and CSFV, no replication of PhoPeV was detected in the DNAJC14 knock out cell line SK6 DNAJC14-KO. Only APPV infection could be detected in this knockout cell line. Viral antigens were stained with Cy3-conjugated secondary antibodies (red), and cell nuclei were stained with DAPI (blue; lower left corner). All mock controls tested negative.

To investigate whether RNA replication of PhoPeV depends on DNAJC14, porcine DNAJC14 knockout cells (SK6 DNAJC14-KO), as well as DNAJC14-KO rescue cells (SK6 DNAJC14-KO GST-Jiv90-WT), were infected with PhoPeV. For comparison, the pestiviruses BuPV, CSFV Alfort-T, and APPV were included. Replication of PhoPeV, BuPV, and CSFV Alfort-T was detected in SK6 WT cells and in the SK6 DNAJC14-KO rescue cell line expressing GST-Jiv90-WT but not in the SK6 DNAJC14-KO cells (Figure 4). The infection rate of PhoPeV and CSFV Alfort-T was lower in the SK6 DNAJC14-KO rescue cells compared to the wild-type SK6 cells. For BuPV, no difference in viral replication efficiency was detected in wild-type SK6 and in the SK6 DNAJC14-KO rescue cells, which confirms previously described data [29]. In contrast to other ncp pestiviruses, replication of APPV was detected in the SK6 DNAJC14-KO cells, as previously reported [30]. Taken together, the results of our study show that viral replication of PhoPeV is DNAJC14-dependent.

## 4. Discussion

After the discovery of CSFV, BVDV, and BDV in the late 19th and in the middle of the 20th century, the host range of these and other pestiviruses described until the end of the first decade of the 21st century remained limited to members of the order *Artiodactyla* [43]. The identification of pestivirus sequences in a growing number of animal species from bats, rodents, pangolins, and the more recent discovery of PhoPeV in harbor porpoises has expanded the host range of pestiviruses to the orders *Chiroptera*, *Rodentia*, *Pholidota*, and *Cetacea*. So far, virus infections with PhoPeV have only been described in the North and Baltic Sea populations of harbor porpoises [5,6]. However, it appears reasonable to assume that occurrence of this virus is geographically more widespread. While no specific pathological findings were associated with the detection of PhoPeV in samples from dead adult animals, the effects of intrauterine infection with PhoPeV remain unknown. An important feature of pestiviruses like, e.g., BVDV, is the establishment of persistent infections. Persistently infected animals shed the virus for their entire life and are therefore important in virus transmission and spread to susceptible hosts [2,10,27]. Persistent infections of harbor porpoises could also play a significant role in the spread of PhoPeV but have not been described thus far.

To expand our knowledge regarding PhoPeV, the virus was characterized applying different cell culture systems and methods. For comparison, the pestiviruses CSFV and BuPV were included in these analyses. Although PhoPeV is genetically more closely related to BuPV than to CSFV, its in vitro cell tropism is comparable to that of CSFV. While PhoPeV was able to infect porcine, bovine, ovine, and feline cell lines, other mammalian cell lines were not susceptible to PhoPeV (Table 2). For BuPV, a broader in vitro host range was confirmed [31]. The present study showed that the seal cell line SEK-2B was not permissive for PhoPeV, which is in agreement with the absence of PhoPeV or a closely related pestivirus in samples from seals so far. Recently, 277 samples from harbor, gray, and ringed seals that were collected in the Baltic Sea region between 2002 and 2019 tested negative for PhoPeV genome [6]. Interestingly, the seal SEK-2B cells supported BuPV replication, which expands the previously described broad host cell tropism of BuPV [31]. Nevertheless, a broader spectrum of marine mammalian species, especially species of the suborder *Odontoceti*, needs to be investigated to enhance our knowledge of the host range of PhoPeV. The results of the present study show that PhoPeV replicates most efficiently in porcine SK6 cells, while lower titers and lower intracellular RNA copy numbers were detected in the porcine cell line PK-15, as well as in bovine (MDBK), ovine (SFTR), and feline (CRFK) cells. It is known that SK6 cells cannot be stimulated to produce type 1 IFN [15]. Accordingly, it can be speculated that the lack of a type 1 IFN-mediated immune response contributed to enhanced replication of PhoPeV in SK6 cells.

The bovine surface protein CD46 has been described to be significantly implicated in the viral entry of BVDV [40] and other ruminant pestiviruses [44]. While porcine CD46 serves as entry factor for APPV, it is not essential for the entry of CSFV and BuPV [35]. To address the question whether infection of porcine cells with PhoPeV is dependent on CD46, the porcine lymphoma cell line 38A_1_D, which lacks the CD46 surface protein [35], was infected with PhoPeV. Similar to CSFV and BuPV, PhoPeV was able to infect 38A_1_D cells (Figure 2). This suggests that porcine CD46 is not essential for cell entry and replication of PhoPeV in porcine cells. Further infection experiments with PhoPeV using CD46 knockout and complemented cell lines will be useful to conclude the analysis of the role of CD46 in cell entry of PhoPeV.

Unlike all other known pestiviruses, PhoPeV lacks the viral autoprotease N^pro^. One important function of the N^pro^ protein is the inhibition of the activation of IFN α/β genes by proteasome-dependent degradation of IRF3 [2,18]. Due to the lack of host-specific whale epithelial and immune cell lines, established porcine cell systems were used to investigate PhoPeV’s interaction with innate immunity, similar to studies on CSFV and BuPV [42,45]. Consistent with the loss of N^pro^, no inhibition of the IRF3-dependent type 1 IFN signaling pathway was observed in PhoPeV-infected PK-15 cells (Figure 3). Ruggli and co-workers described comparable results by infection of different cell lines with genetically engineered CSFV strains that either lack the entire N^pro^ (CSFVΔN^pro^) gene or the IRF3-binding domains in the N^pro^ protein encoding region [15]. Low virus titers and RNA genome copy numbers in porcine, bovine, ovine, and feline cell lines after infection with PhoPeV may be the result of efficient induction of the innate immune response, as shown for a genetically modified CSFV strain lacking the N^pro^ encoding region in the viral genome (CSFVΔN^pro^) [15]. On the other hand, SK6 cell deficiency in type 1 IFN production [15] can explain the high virus titers after infection with PhoPeV. In contrast to SK6 cells, infection of PK-15 cells with PhoPeV resulted in the expression of ISGs such as Mx1 (Figure 3B). It has been speculated that the loss of the N^pro^ coding region in the genome of PhoPeV could be caused by an adaptation of the virus to the cetacean organism [5]. In addition to N^pro^, the glycoprotein E^rns^ counteracts the induction of type 1 IFN and expression of ISGs by degradation of viral RNA [16,46]. Hereby, E^rns^ is able to bind and cleave viral ss and dsRNA. Based on this, viral genomic RNA is cleaved before detection by pattern recognition receptors (PRRs) and therefore E^rns^ inhibits the initial innate immune response upon infection and viral replication [16,20,21]. Comparison of the E^rns^ aa sequence of different pestivirus species showed that the two conserved RNase activity sites are similar but also that PhoPeV is the only pestivirus that contains a unique asparagine residue in one of the two active sites of the RNase [16]. By comparison of the RNase activity of purified E^rns^ proteins from different pestiviruses, the E^rns^ of PhoPeV exhibited a lower RNase efficiency compared to BuPV and other pestiviruses [16]. It remains to be investigated whether PhoPeV counteracts the type 1 IFN-dependent innate immune response by degradation of viral dsRNA via glycoprotein E^rns^. The possible evolutionary adaptation of PhoPeV to the immune system of cetaceans might be caused by multiple factors. As shown by the infection of specific-pathogen-free pigs with wild-type and ΔN^pro^ mutant CSFV strains, the N^pro^-induced impairment of the IRF3-dependent IFN pathway is not a major factor for pestiviral virulence [41]. Interestingly, it was observed that in *Odontoceti*, the Mx1 and Mx2 genes are dysfunctional [47]. This could make N^pro^ expression dispensable in those hosts. Accordingly, it can be hypothesized that E^rns^ may be sufficient to counteract the innate immune response, which may have led to the loss of the N^pro^ coding region.

For BVDV, CSFV, BuPV, and several other pestiviruses, it has been shown that the cellular protein DNAJC14 plays an important role in the control of pestiviral RNA replication and is significantly implicated in the establishment of persistent infections [29,30]. In contrast, RNA replication of APPV can occur in the absence of DNAJC14 [30]. Experiments using SK6 DNAJC14 knockout and rescue cell lines demonstrated that RNA replication of PhoPeV is dependent on DNAJC14 (Figure 4).

In conclusion, the results of the present study show that in contrast to other ncp pestiviruses, PhoPeV does not degrade IRF3 and, consequently, does not efficiently inhibit a type 1 IFN-mediated innate immune response in PK-15 cells. This unique feature is most likely due to the absence of the complete N^pro^ coding sequence in the viral genome. PhoPeV shows a narrow species-specific cell tropism in vitro that is comparable to that of CSFV. In addition, PhoPeV is able to replicate in a CD46-deficient cell line, suggesting that CD46 is not essential for the entry and replication of PhoPeV. Like most ncp pestiviruses, the cellular protein DNAJC14 is important for the control of PhoPeV RNA replication. Establishment of a harbor porpoise cell line may be useful to expand the knowledge about the interaction of PhoPeV with the innate immune response of its natural host.

## Figures and Tables

**Table 1:** Cell lines used in the present study.

Cell Line	Species	Organ	Medium	Source/Reference
SK6	Domestic pig	Kidney	MEM * + 10% FBS *^1^	Institute of Virology, Mittelhäusern, Switzerland
SK6 DNAJC14-KO	Domestic pig	Kidney	MEM + 10% FBS	Institute of Virology and Cell Biology, University of Lübeck, Germany
SK6 DNAJC14-KO GST-Jiv90-WT	Domestic pig	Kidney	MEM + 10% FBS	Institute of Virology and Cell Biology, University of Lübeck, Germany
PK-15	Domestic pig	Kidney	MEM + 5% FBS	Institute of Virology, Hannover, Germany
38A_1_D	Domestic pig	Lymphoma	MEM + 10% FBS	Institute of Virology, Hannover, Germany
MDBK	Cattle	Kidney	EDulb *^2^ + 10% HS *^3^	Rockville, MD, USA
SFTR (CCLV-RIE 43)	Domestic sheep	Thymus	EDulb + 10% FBS	CCLV, Friedrich-Loeffler-Institute, Island Riems, Greifswald, Germany
SEK-2B	Seal	Kidney	EDulb + 10% FBS	Institute of Virology, Gießen, Germany
CRFK	Cat	Kidney	EDulb + 10% FBS	Institute of Virology, Hannover, Germany
Vero	Vervet monkey	Kidney	EDulb + 10% FBS	Institute of Virology, Hannover, Germany
BHK-21	Golden hamster	Kidney	EDulb + 10% FBS	Institute of Virology, Hannover, Germany
Balb3T3	Mouse	Embryo	EDulb + 10% FBS	Institute of Virology, Hannover, Germany
HEK-293T (ACC-635)	Human	Embryonic Kidney	EDulb + 10% FBS	DSMZ, German Collection of Microorganisms and Cell Cultures, Braunschweig, Germany
HeLa	Human	Cervix carcinoma	EDulb + 10% FBS	Institute of Virology, Hannover, Germany

* MEM = minimum essential medium; *^1^ FBS = fetal bovine serum; *^2^ EDulb = Dulbecco’s Modified Eagle’s Medium; *^3^ HS = horse serum.

**Table 2:** Susceptibility of mammalian cells for infection with PhoPeV, BuPV, and CSFV Alfort-T.

Cell Lines	Viruses		
	BuPV	PhoPeV	CSFV Alfort-T
SK6	+	+	+
PK-15	+	+	+
38A_1_D	+	+	+
MDBK	+	+	+
SFTR	+	+*	+
CRFK	+	+	+*
SEK-2B	+	-	-
BHK-21	-	-	-
Vero	+	-	-
HeLa	+	-	-
HEK-293T	+	-	-
Balb3T3	-	-	-

+ = susceptible; - = not susceptible; * = not able to produce infectious particles to infect the same cell line.

## Data Availability

The data sets are submitted together with the study.
